# Regulation of Epithelial Branching Morphogenesis and Cancer Cell Growth of the Prostate by Wnt Signaling

**DOI:** 10.1371/journal.pone.0002186

**Published:** 2008-05-14

**Authors:** Bu-Er Wang, Xi-De Wang, James A. Ernst, Paul Polakis, Wei-Qiang Gao

**Affiliations:** 1 Department of Molecular Biology, Genentech, Inc., South San Francisco, California, United States of America; 2 Department of Protein Chemistry and Protein Engineering, Genentech, Inc., South San Francisco, California, United States of America; 3 Department of Cancer Target and Pathways, Genentech, Inc., South San Francisco, California, United States of America; Centre de Regulacio Genomica, Spain

## Abstract

Although Wnt signaling has been shown to be important for embryonic morphogenesis and cancer pathogenesis of several tissues, its role in prostatic development and tumorigenesis is not well understood. Here we show that Wnt signaling regulated prostatic epithelial branching morphogenesis and luminal epithelial cell differentiation in developing rat prostate organ cultures. Specifically, Wnt signaling regulated the proliferation of prostate epithelial progenitor cells. Assessment of the expression levels of a Wnt pathway transcriptional target gene, Axin2, showed that the Wnt pathway was activated in the developing prostate, but was down-regulated in the adult. Castration resulted in an upregulation of Axin2 whereas androgen replacement resulted in a down regulation of Axin2. Such dynamic changes of Wnt activity was also confirmed in a BAT-gal transgenic mouse line in which β-galactosidase reporter is expressed under the control of β-catenin/T cell factor responsive elements. Furthermore, we evaluated the role of Wnt signaling in prostate tumorigenesis. Axin2 expression was found upregulated in the majority of human prostate cancer cell lines examined. Moreover, addition of a Wnt pathway inhibitor, Dickkopf 1 (DKK1), into the culture medium significantly inhibited prostate cancer cell growth and migration. These findings suggest that Wnt signaling regulates prostatic epithelial ductal branching morphogenesis by influencing cell proliferation, and highlights a role for Wnt pathway activation in prostatic cancer progression.

## Introduction

The Wnt signaling pathway is crucial in a variety of biological process including neural patterning, planar polarity, stem cell maintenance and cell differentiation [1]. This has been demonstrated in a number of systems, via genetic and biochemical approaches. The Wnt signaling is initiated by extracellular proteins, i.e., Wnts, through binding to their respective frizzled family of receptors. The signal is then transduced to β-catenin via a cascade of signal transducing molecules in the cytoplasm. β-catenin then enters into the nucleus, forms a complex with TCF and transactivates the downstream target genes that regulate or participate in various processes. The complexity of signaling arises in part from the multitude of components in this pathway including for example 19 human Wnt ligands, 1 Wnt inhibitory factor (WIF), 5 secreted frizzled related proteins (SFRPs) that can sequester Wnt ligands from binding to their cognate receptors, 2 low density lipoprotein receptor-related proteins (LRP5/6) and 4 Dickkopf (Dkk) proteins that modulate the activity of frizzled receptors [1], [2].

In the canonical Wnt pathway, stabilization and translocalization of β-catenin to the nucleus is a critical step of Wnt signaling activation. In absence of Wnt ligand binding, β-catenin is targeted to degradation via its interaction with the adenomatosis polyposis coli (APC) protein and the Wnt signaling is minimally activated. When APC is mutated, β-catenin accumulates in the nucleus and Wnt signaling is constitutively active [1]. The Wnt signaling downstream program is poorly understood, and a very useful downstream gene specific for this pathway is Axin 2, which upon transcription and translation, acts as a negative feedback regulator of the Wnt siginaling pathway, by helping direct β-catenin for degradation in the proteasome [3], [4]. Stabilization of β-catenin protein and elevation of Axin2 transcript are considered indicators of Wnt pathway activation [4].

Dysregulation of Wnt signaling is also associated with cancer pathogenesis of various tissues [5], [6]. Genetic alterations in the APC genes causes predisposition for colorectal cancer (Groden, Cell 1991), and the tumor suppressor function of APC/oncogenic effect of Wnt pathway is clearly demonstrated in APCmin transgenic mice where numerous adenomas form in the intestine [7]. The role of Wnt pathway in prostate tumorigenesis has recently received increased attentions but is still not well understood. Recent studies reported that certain Wnt ligands such as Wnt1 are expressed in prostate cancer cell lines and appear to be elevated in some human prostate tumor tissues [8]. Specific Wnt pathway inhibitors such as WIF1 appear to be down-regulated in a considerable percentage of prostate cancer samples [9]. Prostate-specific deletion of APC gene in mice results in formation of adenocarcinoma [10]. In addition, interaction between β-catenin and the androgen receptor (AR) has been shown to enhance AR-mediated transcription [11], which plays a critical role during prostatic cancer progression. Elucidating how Wnt signaling regulates prostatic development and tumorigenesis would facilitate the development of novel therapeutic agents for the treatment of prostate cancer.

In an effort to understand the role of Wnt signaling in prostatic epithelial development, we performed organ cultures of developing prostates prepared from early postnatal rats to determine the effects of modulation of the Wnt signaling pathway on prostatic epithelial branching morphogenesis. We found that Wnt signaling regulated prostatic epithelial cell differentiation through influencing the proliferation of prostatic epithelial progenitor cells. In addition, TaqMan RT-PCR analysis revealed that several commonly studied prostate cancer cell lines and xenografts exhibited activation of the Wnt pathway. Moreover, Dickkopf1 (DKK1), a Wnt pathway inhibitor [12]–[14], significantly inhibited prostate cancer cell growth and migration. These findings together suggest that activation of the Wnt pathway plays a crucial role during prostatic development, and regrowth and that inhibiting the Wnt pathway might be of therapeutic value in the management of prostate cancer progression.

## Results

### Wnt signaling regulates prostatic epithelial branching morphogenesis

**Figure 1:**
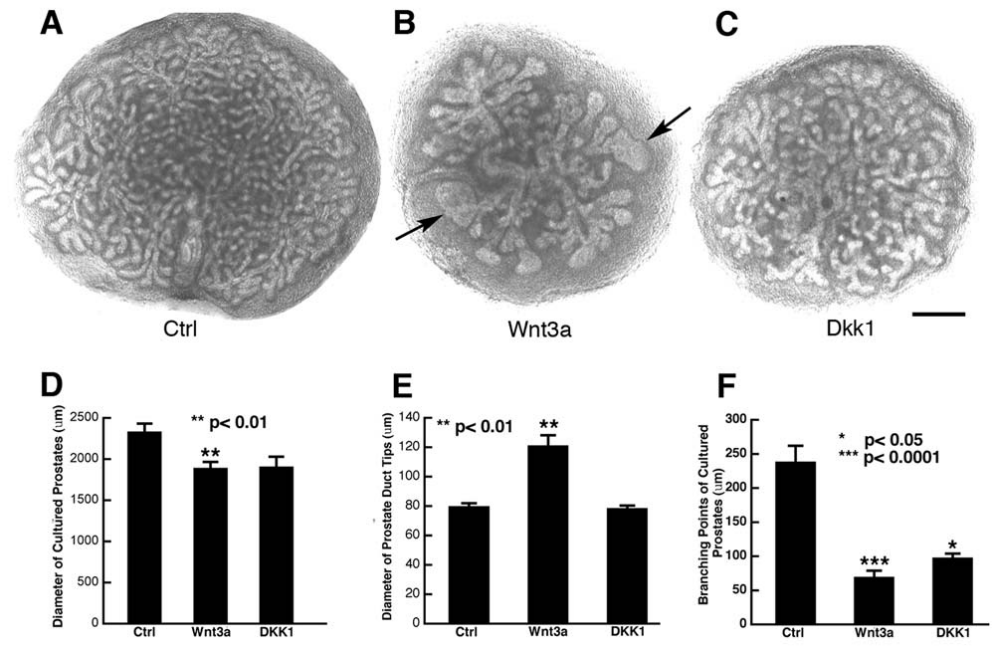
Wnt3a and DKK1 regulate prostatic epithelial branching morphogenesis. Whole mount ventral prostates were prepared from P2 rats and maintained for 7 days in serum-free medium in the absence (A) or presence of 50 nM of Wnt3a (B) or 400 nM of DKK1 (C). Similar patterns were consistently seen in 3 repeat experiments of 5–6 prostate organs per group in each individual experiment. Note that addition of either Wnt3a or DKK1 to the culture resulted a change in epithelial branching morphogenesis. Quantification of the cultures by measuring the diameter of the cultured prostates (D) and the ductal tips (E) using Axiovision software and the branching points (F) were done by analyzing 4 randomly selected cultures per group. Data are expressed as mean + SEM (t-test, compared to control cultures). Bar, 400 µm.

Prostatic epithelial branching morphogenesis in the rodent occurs mainly after birth by expansion and further branching from the epithelial primodia [15], [16]. This process can be recapitulated in a convenient organ culture system [17], [18]. To examine whether Wnt signaling plays a role in prostatic epithelial branching morphogenesis, we treated organ cultures of postnatal day 2 (P2) rat ventral prostates with a Wnt ligand, Wnt3a, or a potent Wnt signaling inhibitor, DKK1 [12]. As shown in Fig. 1, the prostate in control cultures showed extensive branching with not only primary and secondary ducts but also tertiary, fine branches after 7 days in culture (Fig. 1A). However, the tissue in the cultures treated with Wnt3a at a concentration of 50 nM displayed blunted, enlarged ductal tips (arrow in Fig. 1B) and a reduced number of tertiary, fine branches. Prostates treated with DKK1 at 400 nM, the prostates appeared smaller in size and had fewer epithelial branches. However, in contrast to Wnt3a-treated prostates, DKK1 treated prostates lacked enlarged ductal tips (Fig. 1C). Quantification of these cultures by measuring the diameters of the cultured prostates and the ductal tips and counting the branching points of these cultures showed statistical differences between the control cultures and the cultures treated with Wnt3a in all 3 aspects (Fig. 1D-F). These results suggest that both activation and inhibition of Wnt signaling affect adversely prostatic epithelial branching morphogenesis, and highlight the importance of precisely-regulated Wnt signaling for the proper development of the prostate.

### Wnt signaling influences cell proliferation and differentiation in the developing prostate epithelium

**Figure 2:**
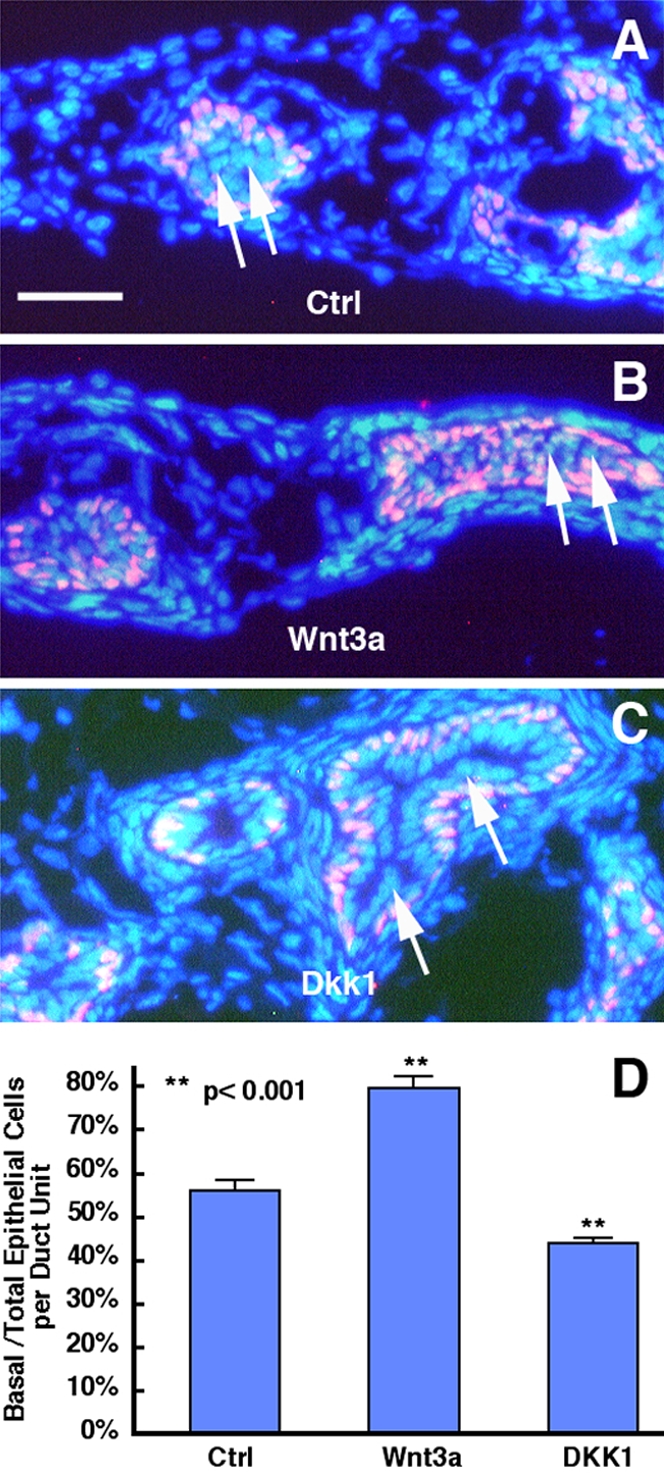
Wnt signaling prevents prostatic epithelial cell differentiation. (A,B,C) p63 immunocytochemistry (red) of the P2 rat ventral prostate organ cultures maintained for 7 days in the absence (A) or presence of 50 nM of Wnt3a (B) or 400 nM of DKK1 (C). The tissue sections were counterstained with DAPI (blue). While p63 positive cells (purple) represent basal cells where progenitor cells reside, the blue cells (arrows) that are negative to p63 in the epithelium are differentiated luminal cells. (D) Quantification of p63 positive cells over total epithelial cells. Data were collected from randomly selected 22–27 ductal units from sections of the organ cultures per group and are expressed as mean + SEM (t-test). Note that while Wnt3a led to a significant increase in the number of basal cells, DKK1 resulted in a reduction in basal cells. Bar, 50 µm for A-C.

Prostatic epithelium is mainly composed of basal and luminal cells along with a minor population of neuroendocrine cells [19]. The basal cells express p63 [20], cytokeratin 14 (CK14) and CK5 and are believed to contain the progenitor cell population [21]. The luminal cells express CK8 or CK18, and are generally post-mitotic and terminally differentiated cells. In rodents, nearly all epithelial cells are progenitor cells and are proliferating until P5 when some of the progenitors undergo terminal mitosis and differentiate into luminal cells that express only CK8 or CK18 [19]. To determine if modulation of Wnt signaling influences the differentiation of progenitors into luminal cells, we performed immunohistochemistry on sections of the prostate organ cultures using basal and luminal cell markers, p63 and CK8, respectively. As shown in Fig. 2, more p63 positive cells were seen in the ductal region of Wnt3a-treated cultures (Fig. 2B) than the control cultures (Fig. 2A). In contrast, fewer p63 positive cells were present in DKK1-treated cultures (Fig. 2C). The cells that were labeled by DAPI nuclear staining in the epithelium, but negative for p63, represent those differentiated luminal cells, which was confirmed by CK8 immunostaining (data not shown). Cell counts from randomly selected cultures of the 3 groups revealed that the percentage of basal cells over the total epithelial cell population within a given individual ductal unit was significantly higher in the Wnt3a-treated cultures as compared with control cultures (Fig. 2D). In contrast to Wnt3a action, the ratios of basal cells vs. total epithelial cells were significantly lower in DKK1-treated cultures than that in control cultures (Fig. 2C). Immunostaining using a CK8 antibody showed a complementary pattern: Wnt3a resulted in a reduction in the number of luminal cells whereas DKK1 led to an enhanced number of luminal cells (data not shown). The change in basal vs. total epithelial cells were not attributable to cell death as TUNEL labeling only detected minimal, background signals with no difference among the three groups of cultures (data not shown).

**Figure 3:**
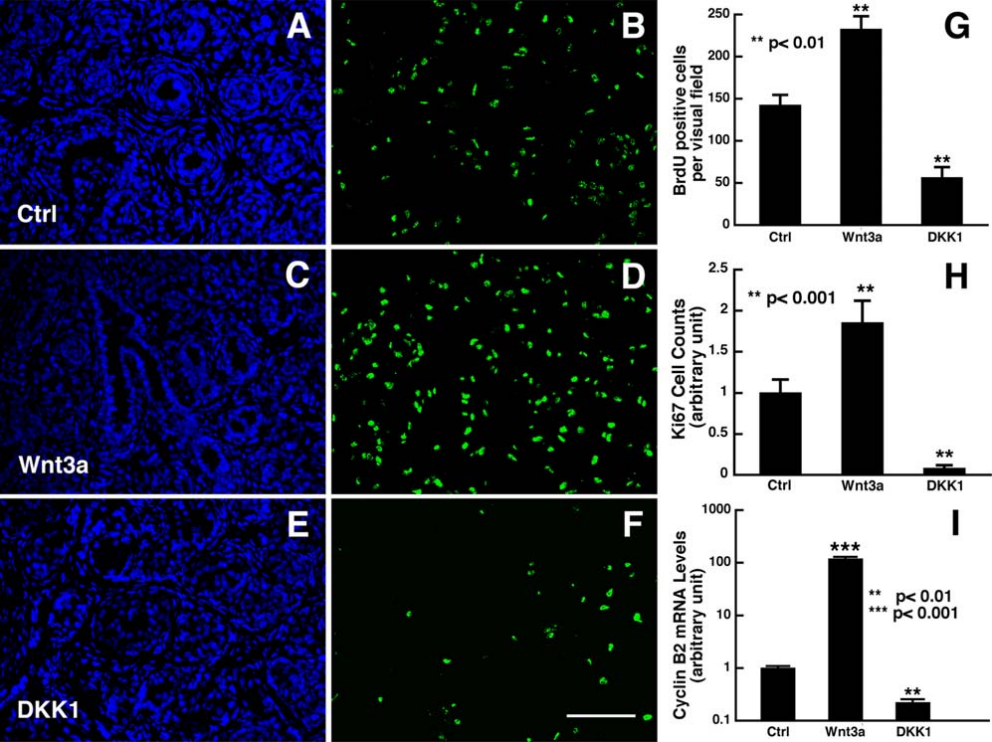
Activation of Wnt signaling enhances prostate epithelial cell proliferation. (A-F) Shown are anti-BrdU antibody (B,D,F) and DAPI (A,C,E) double labeling of the P3 rat ventral prostate organ cultures maintained for 3 days in the absence (A,B) or presence of 50 nM of Wnt3a (C,D) or 400 nM of DKK1(E,F). (G). Quantification of BrdU-positive cells in a given visual field of 434 µm×322 µm. (H) Quantification of Ki67-positive cells in the P2 prostate cultures maintained for 7 days, which was normalized to the control cultures. Cell counts (for G and H) were performed from randomly selected visual fields of 5 organ cultures per group and data were expressed as mean+SEM (t-test). Note that while Wnt3a significantly enhanced progenitor cell proliferation, DKK1 inhibited progenitor cell proliferation. (I). Expression level change of cyclin B2 in P2 rat prostate organ cultures.. Data were collected from 4 organ cultures maintained for 2 days per group and are expressed as mean+SEM (t-test). Note that expression of cyclin B2 was upregulated by wnt3a, but down-regulated by DKK1. Bar, 100 µm for A-F.

Given that Wnt3a and DKK1 treatments led to an enhanced and decreased number of p63 positive cells, respectively, which are mainly composed of progenitors at this early developmental stage, we sought to determine whether Wnt3a and DKK1 would affect the proliferation of progenitor cells in these prostate organ cultures using bromo-deoxyuridine (BrdU) and Ki67 immunohistochemistry. As shown in Figure 3, after a 3- day treatment, more proliferating cells were observed in cultures treated with Wnt3a (Fig. 3C,D), and fewer proliferating cells in cultures treated with DKK1 (Fig. 3E,F), as compared to the control cultures (Fig. 3A,B). Cell counts performed from randomly selected fields indicated that there was a 1.63-fold increase in the number of Ki67 positive cells in Wnt3a-treated prostate organs compared to control cultures (Fig. 3G). In contrast, there was a significant decrease in the number of Ki67 positive cells in DKK1-treated prostate organs (Fig. 3G). Consistent with the BrdU incorporation assays, our Ki67 immunohistochemistry in the 7-day cultures also showed similar cell proliferation-enhancing and inhibiting effects by Wnt3a and DKK1, respectively (Fig. 3H). To understand how cell proliferation is regulated by Wnt signaling during prostate development, we examined expression patterns of several cyclin genes in a separate microarray study of developing mouse prostates. We found that cyclin B2 expression was significantly higher at P4 than P19 and 10 weeks old mice (approximately 4- and 8-fold, respectively) and that the association of cyclin B2 with early development was much stronger than those of other cyclins (data not shown). To confirm whether the elevated proliferation, based on Ki67 staining, seen in the organ culture might be partly mediated by cyclin B2, we performed TaqMan RT-PCR analysis of these cultures. As shown in Fig. 3I, the expression of cyclin B2 was approximately 118.8-fold higher and about 4.5-fold lower in the organ cultures treated with Wnt3a and DKK1, respectively. Taken together, these findings suggest that Wnt signaling regulates the terminal differentiation of basal cells into luminal cells by controlling the proliferation and/or maintenance of epithelial progenitor cells.

### Dynamic changes of Wnt signaling activation during prostatic development, after castration and following androgen replacement

**Figure 4:**
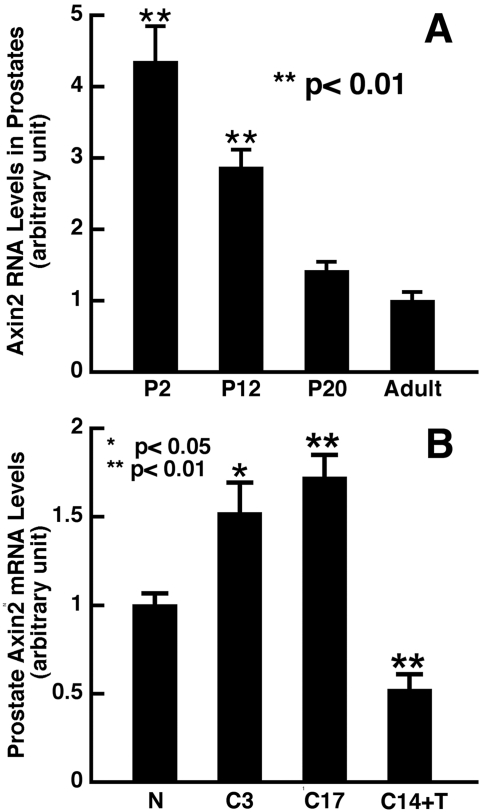
TaqMan RT-PCR analyses of Axin2 expression during prostate development and regrowth following androgen deprivation and replacement. (A) A gradual downregulation of Axin2 from newborn to adulthood.nbsp;(B) Axin2 was upregulated following castration, but returned to normal low levels after androgen replacement. Data were collected from 4–6 samples per group and are expressed as mean+SEM (t-test, compared to normal adult prostates). Abbreviation: N, normal prostate; C3, 3 days after castration, C17; 17 days after castration; C14+T. 14 days after castration+3 days of testosterone treatment.

To provide further supportive evidence for the role of Wnt signaling in the maintenance of prostate epithelial progenitors, we performed quantitative RT-PCR using Axin2 as an indicator for activation of Wnt signaling with prostate tissue prepared at various time points of development. We found that Axin2 levels were the highest at P2 but declined over time as the prostate matured (Fig. 4A). These data suggest that Wnt signaling is more active at the early stages of developing prostates, consistent with a higher progenitor cell population than in fully developed prostates where the majority of the epithelial cells are terminally differentiated luminal cells.

Previous studies have shown that the portion of the basal cell compartment over the total epithelial cells in the prostate epithelium is altered following castration and hormonal replacement [22]. Castration-induced androgen withdrawal is known to deplete approximately 90% of the epithelial content [23] and results in enrichment of the basal/progenitor cell population. Androgen replacement in turn leads to proliferation of the progenitor cells and their differentiation into luminal cells, resulting in prostate re-growth back to its normal size [24]. We performed quantitative real-time RT-PCR to compare expression levels of Axin2 in prostates harvested from normal mice, mice after 3 days and 17 days following castration and mice after 3 days of testosterone replacement following castration. As shown in Fig. 4B, Axin2 levels were 1.5-fold and 1.7-fold higher in prostates 3 and 17 days following castration, respectively, but declined 3 days after testosterone replacement. These data from both developing and regrowing prostates indicate that Wnt signaling is positively correlated to basal cell number and inversely correlated with differentiation of progenitors into luminal cells in prostates.

**Figure 5:**
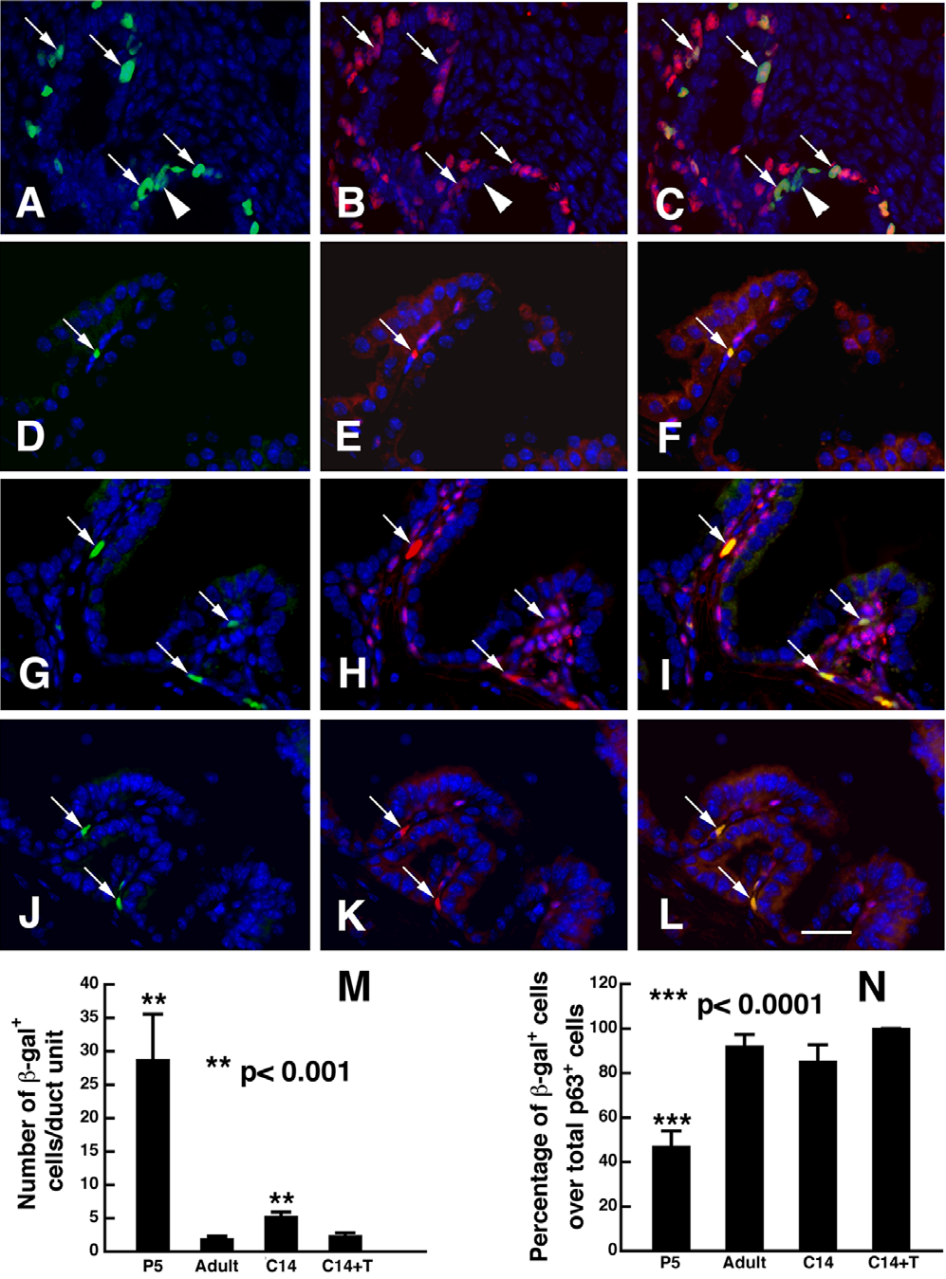
β-galactosidase expression in BAT-gal transgenic prostates. Double immunostaining of prostate tissue sections with anti-β-galactosidase (green in A, D, G, J) and anti-p63 (red, in B, E, H, K) antibodies, prepared from mice of P5 (A-C), adult (D-F), 14 days post-castration (G-I) and 14 days after androgen replacement (J-L). The sections were counterstaining with DAPI (blue). While arrows indicate cells that co-express p63 and β-galactosidase, arrowhead (Fig. 1A-C) shows a cell that is β-gal positive but p63 negative in the developing prostate. Note that β-galactosidase-expressing cells are located exclusively in the epithelium, and mainly within the basal cell compartment in the adult prostate, although a noticeable number of the are seen in the luminal cell layer in developing prostate. Bar, 50 µm. (M) Quantification of β -gal positive cells per epithelial ductal unit, which is determined in the tissue sections based on DAPI counterstaining and the presence of an epithelial lumen. (N) Cell counts of β -gal positive cells over total basal cells (p63 positive cells). Data were collected from 14–20 randomly selected ductal units in the sections from 4–5 prostates per group and are expressed as mean+SEM (t-test, compared to normal adult prostates).

We next examined a BAT-gal transgenic mouse line in which β-galactosidase reporter is expressed under the control of β-catenin/Tcell factor responsive elements [25]. This mouse line has been shown to be *bona fide* in vivo indicators of Wnt/β-catenin signaling, allowing visualization of the general status of Wnt activation in cells in a given tissue. Examination of tissue sections prepared from prostates at different stages including P5 (developing prostates, Fig. 5A-C), adult (mature prostates, Fig. 5D-F)), post-castration (Fig. 5G-I) and following androgen replacement (Fig. 5J-L), indicated that the number of β-galactosidase-positive cells was much higher in developing prostates, than adult prostates (28.7±6.9/duct, n = 10 vs. 1.92±0.4/duct, n = 13, Fig. 5M). Castration led to an elevated number of β-galactosidase-positive cells (5.2±0.7/duct, n = 13), but androgen replacement resulted in a lower number that was equivalent to the normal level in adult prostates (2.3±0.5/duct, n = 15, Fig. 5M). In addition, We found that Wnt activity was exclusively seen in epithelial cells, mainly in basal cell compartment where progenitor cells reside. In addition, double labeling of these tissue sections with anti-p63 antibody, a basal cell marker, and anti-β-galactosidase antibody revealed that in the adult, the vast majority of β-galactosidase-positive cells were co-labeled with anti-p63 antibody (arrows in Fig. 5A-L and N), although in postnatal prostates, there were noticeable number of β-galactosidase-positive cells in luminal cell layer, which could be due to a possibility of delayed degradation of β-galactosidase activity in the terminally mitotic cells that exited cell cycle to differentiate into luminal cells. Therefore, these findings not only confirm the dynamic changes of Wnt activity during prostate development and regrowth obtained by TaqMan RT-PCR analysis of Axin2 expression, but also provide additional support for association of Wnt signaling with prostate basal cells where progenitor cells are generally believed to reside.

### Inhibition of Wnt signaling leads to suppression of cell proliferation and migration of prostate cancer cells

**Figure 6:**
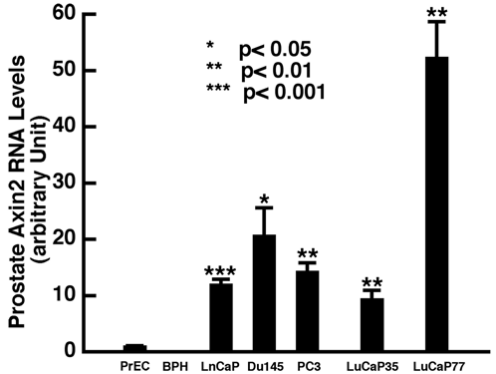
Wnt signaling is activated in prostate cancer cells. TaqMan RT-PCR analysis of Axin2 expression in various human prostate epithelial cells and cancer cells. Data were collected from triplets and are expressed as mean+SEM (t-test). Note that Axin2 expression is much higher in prostate cancer cell lines (LNCaP, Du145, PC3) and human prostate tumor xenografts (LuCaP35, LuCaP77) as compared to primary prostate epithelial cells (PrEC) or non-tumorigenenic immortalized prostate epithelial cells (BPH1).

Aberrant Wnt signaling has been observed in many tumors [5], [6]. To determine whether Wnt signaling plays a role in prostatic tumorigenesis and tumor progression, we assessed the Wnt signaling status in human prostate cancer cell lines and 2 human prostate tumor xenografts by measuring expression levels of Axin2. As shown in Fig. 6, quantitative RT-PCR analysis revealed that Axin2 was expressed at higher levels in PC3, DU145 and LNCaP cell lines and human prostate tumor xenograft samples (LuCaP35 and LuCaP77) [26], than normal or non-tumorigenic human prostate epithelial cells such as PrEC and BPH1 cells [27]. Hence, Wnt signaling is upregulated in prostate cancer cells compared to normal prostate epithelial cells.

**Figure 7:**
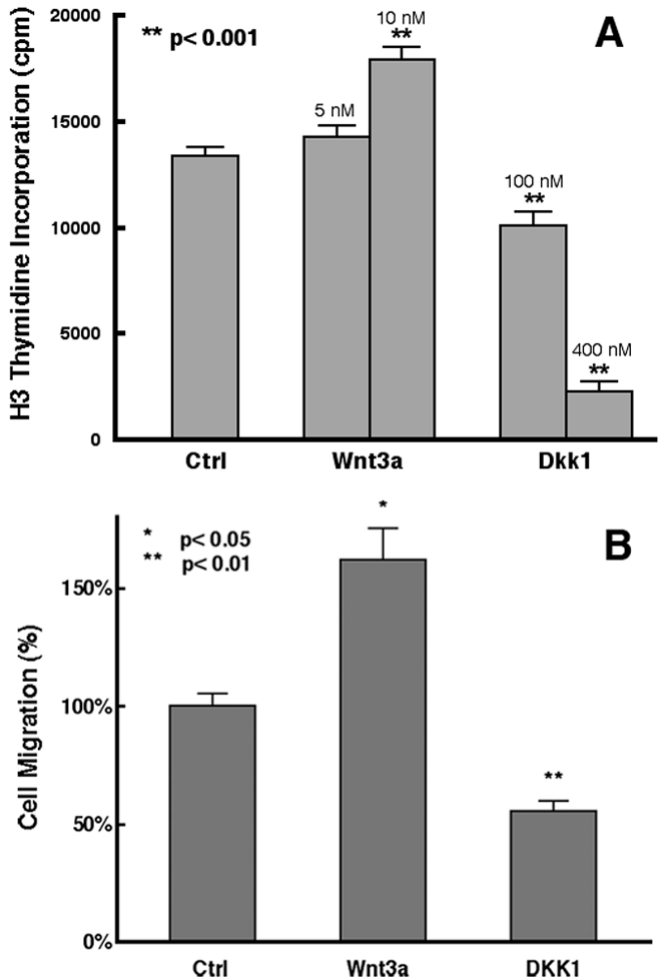
DKK1 inhibits proliferation and migration of prostate cancer cells. (A) Tritiated thymidine incorporation in PC3 cultures in the absence or presence of Wnt3a or DKK1. While high concentration of Wnt3a (10nM) enhanced PC3 cell proliferation, DKK1 inhibited PC3 proliferation in a dose-dependent manner. (B) Regulation of PC3 cell migration by Wnt signaling. Data were collected from 6–8 cultures per group and are expressed as mean+SEM (t-test). Note that Wnt3a increased the number of migrated cells, whereas DKK1 inhibited cell migration.

We next assessed whether Wnt3a or DKK1 treatment would affect the growth of PC3 cells. By measuring cell proliferation using ^3^H thymidine incorporation, we found that treatment of the cultures with Wnt3a resulted in a significant increase in cell proliferation (Fig. 7A). In contrast, DKK1 treatment reduced cell proliferation in a dose-dependent manner (Fig. 7A). Similar results were also observed in LNCaP cells (data not shown).

To examine whether Wnt signaling also affects prostate cancer cell migration or cell motility, we performed cell migration experiments with PC3 cells. As shown in Fig. 7B, addition of Wnt3a into the culture medium led to an enhanced number of migrated cells, whereas DKK1 inhibited cell migration, compared to the control culture. In contrast, treatment of BPH1 cells in which expression Axin2 was low or not detectable (Fig. 6) with DKK1, no inhibitory effects on cell migration was seen (data not shown). On the other hand, either trypan blue staining or FACS sorting using annexin 5 immunostaining did not reveal enhanced cell death in the DKK1-treated cultures (data not shown). These results suggest that Wnt signaling contributes to increased cell motility, which in turn may promote the invasiveness and/or metastasis of prostate cancer cells.

## Discussion

### Role of Wnt signaling during prostate development

In this study, we investigated the role of Wnt signaling pathway in the prostate first from a developmental biology perspective and then as an extension, in a closely related pathogenic process, i.e, tumorigenesis. The *ex vivo* postnatal prostatic tissue culture experiments not only allows convenient monitoring of the effects of growth factors or inhibitory agents on early prostate development, but also enables conducting the experiments even when the supply of reagents is fairly limited. This system has been used previously in elucidating the role of androgenic hormones, tissue components and various novel pathways in prostatic branching morphogenesis and early development [17], [18], [28]–[30]

We have directly examined the role of Wnt signaling during prostate development by using both Wnt signaling stimulating and inhibitory factors, Wnt3a and DDK1, respectively. Addition of the Wnt ligand, Wnt3a, results in enhanced cell proliferation and a reduction of luminal epithelial cell differentiation, whereas treatment with the Wnt pathway inhibitor, DKK1, enhanced cell differentiation and reduced cell proliferation. Modulation of cell proliferation by Wnt signaling can be attributed, in part, to altered levels of cyclin B2, as cyclin molecules are previously reported to play a role in prostate cell proliferation [31], [32]. These data together suggest that the requirement for the Wnt signaling in prostate branching morphogenesis is tightly and delicately regulated as both increased and decreased Wnt activity could adversely affect prostate branching morphogenesis in ex vivo cultures. Furthermore, our analysis of messenger RNA levels of Axin2 and β-galactosidase expression in BAT-gal transgenic mice as indicators of Wnt pathway activity in postnatal prostates also suggest that Wnt pathway is more active in developing prostates than mature prostates.

### Association of Wnt signaling and prostate basal cells where progenitor cells are generally believed to reside

Our findings from organ cultures of developing prostates are consistent with the role of Wnt signaling in maintenance of progenitor cells in other tissues such as the intestine [33], mammary gland [34] and hematopoietic system [35]. In addition, correlation of basal cells where progenitor cells are generally believed to reside with activation of the Wnt pathway is also observed during prostate re-growth following castration and hormonal replacement. Epithelial progenitor cells in the prostate are independent of androgen for survival. Accordingly, previous studies have shown that following castration, the prostate epithelial progenitor cell population is enhanced or enriched [30], [36], [37] due to apoptosis of androgen-dependent, terminally differentiated luminal epithelial cell population. Conversely, when androgen is replaced the prostate is triggered to re-grow to its original size attributable to enhanced proliferation of progenitor cells and their subsequent differentiation into new luminal cells. Using Axin2 as a molecular indicator, we found that Wnt is upregulated following castration but is down-regulated after androgen replacement, corresponding nicely with the content of progenitor cells in the prostate. Similar dynamic pattern of Wnt activity was seen in BAT-gal transgenic mice. Suppression of the Wnt signaling pathway after hormonal replacement, as indicated by the down-regulation of Axin2 mRNA or reduced number of β-galactosidase-positive cells may be due to an interaction between β-catenin and AR pathway. β-catenin has been shown able to interact with AR and functions as a co-activator of AR [38]. It is possible that following ligand binding, the active form of AR, in addition to its normal function as a transcription factor for prostate development, also binds and sequesters nuclear β-catenin, leading to a reduction in the pool of β-catenin available for β-catenin-TCF trans-activation [38]. As a result of reduced Wnt signaling, prostate progenitor cells would then be induced to differentiate into luminal cells. Taken together, these findings provide supportive evidence for the role of Wnt signaling in progenitor cell maintenance in the prostate epithelium.

### Implications of Wnt signaling in prostate cancer cell growth

Increasing evidence suggests that the Wnt pathway may also contribute to prostate cancer progression [39]. Several Wnt ligand including Wnt1 and Wnt2 are up-regulated in human prostate tumor samples [40], [41]. sFRP3 can suppress prostate tumor cell growth and invasion [42]. Dkk3 has recently been shown to be down-regulated in human prostate tumor samples, and DKK1 treatment can inhibit prostate tumor cell growth [43]. More lately, it is reported that adenocarcinomas are formed in a prostate-specific APC deletion mouse model [10]. Our study indicates that Axin2 is elevated in several human prostate cancer cell lines and xenograft human prostate tumors as compared to normal human PrEC and non-tumorigenic immortalized human prostate epithelial cells, providing further support for an oncogenic role for Wnt pathway activation in prostate tumorigenesis. In addition, our finding that DKK1 treatment significantly inhibits prostate cancer cell growth and migration reinforces this notion. Due to inherent difficulties associated with the production and purification of a large quantity of DKK1 required for in vivo xenograft experiments, the in vivo prostate cancer xenograft experiment has not been performed, but is warranted in the future to confirm the inhibitory effects on DKK1 on prostate tumor growth and progression.

A cancer stem cell model has recently been proposed for many cancer types. In the case of prostate cancer, cancer stem cells are those that are believed to be independent of androgen for survival. Given the fact the Wnt signaling is important for cell proliferation and differentiation during normal development and is associated with the basal cells where progenitor cells are generally believed to reside during re-growth following castration and androgen replacement, it would be interesting to determine if Wnt pathway is also associated with prostate cancer stem cells that may play a crucial role in androgen resistance and late stage prostate cancer progression. Isolation of prostate cancer cells that express receptors for Wnt ligands to perform tumorigenicity as well as stem cell property studies would help answer this question and provide insight into whether Wnt signaling is associated with prostate cancer stem cells. If so, this may highlight targeting prostate cancer stem cells as an alternative mechanism of action of Wnt pathway inhibitors in the treatment of prostate cancer.

In summary, to our knowledge, this is the first report to show that Wnt signaling regulates prostatic epithelial branching morphogenesis via influencing cell proliferation and differentiation in the epithelium. Consistent with other studies [10], [11], [40], [41], [43], activation of the Wnt pathway contributes to prostate cancer progression. Inhibition of Wnt pathway might therefore be of therapeutic value in the management of prostate cancer.

## Materials and Methods

### Organ culture of prostate tissue

Ventral prostates were dissected from postnatal day 2 (P2) rats and placed on 8-µm cell culture inserts (BD Biosciences) in serum-free medium containing Dulbecco's modified Eagle's medium/F-12 plus serum-free supplement (I-1884; Sigma), 1% bovine serum albumin, 2 mM glutamine, 5 mg/ml glucose, 25 ng/ml fungizone, and 100 units/ml penicillin and 100 mg/ml streptomycin, as previously [18]. Wnt3a (50 nM, R&D), and DKK1 (400 nM, Genentech, Inc) were added to the serum-free medium in the experimental groups at the beginning of organ cultures. The medium was changed every other day. In some cultures, bromodeoxyuridine (BrdU, Amersham, 1:1000) was added on the third day for 2 hr before the cultures were fixed in 4% paraformaldehyde (30min). The other cultures were fixed in ice-cold methanol for 15 mins at room temperature prior to processing for immunohistochemistry.

### Cell culture, 3H-thymidine incorporation and Migration assays

The PC3 cell line was purchased from ATCC and maintained in Dulbecco's modified Eagle's medium/F-12 plus 10% fetal bovine serum (Sigma). For 3H-thymidine incorporation assays, cells were plated at 4000 cells/well in a 96-well plate in serum free medium with either Wnt3a (5 nM or 10 nM) or DKK1 (100nM or 400 nM) for 24 hours. 3H-thymidine (1 µCi/well) was added for 16 hours, and cells were harvested using a Tomtec cell harvester. Incorporated H3-tymidine was counted with TOP Count (Pachard Instrument Company, Meriden, CT) as described [44]. Each of the experimental groups had 8–12 replicates, and the data were expressed as mean±SEM. A two-tailed, unpaired t test was used for statistical analysis. For migrating assay, cells were plated at 5×10^4^/ml for 24-well chambers (#354480, BD Biosciences) in 0.5 ml of Dulbecco's modified Eagle's medium/F-12 plus 10% fetal bovine serum for 22 hours. The cells on the upper surface of the membrane were removed by scrubbing; the cells on the lower surface of the membrane were fixed with 100% methanol, stained with H & E, photographed and counted.

### Prostate regrowth following hormone replacement

Mice at the age of 12–14 weeks old were castrated as described [30]. Some mice were implanted with testosterone pellets (15 mg/pellet/mouse, Innovative Research, Sarasota, FL) for 3 or 14 days after 14-day post-castration. Mice were euthanized and prostates were harvested at 3 or 17 days after castration. Some of the experiments were also performed with BAT-gal transgenic mice [25]. All animal experiments were performed in accordance with approved guidelines of institutional animal care and use committee at Genentech.

### Immunocytochemistry

Five-micrometer sections of prostate cultures/tissue were stained with anti-p63 antibody (1:200 dilution; Santa Cruz Biotechnology), anti-β−galactosidase (1:10,000, MP Biomedical), anti-BrdU (1:40, BD Biosciences), or Ki67 antibody (1:50 dilution, DAKO, Carpinteria, CA) followed with AlexFluor 594 goat anti-rabbit secondary antibody (Invitrogen. Carlsbad, CA) and/or AlexFluor 488 goat anti-mouse IgG (Invitrogen). The slides were mounted in Fluoromount-G (Southern Biotechnology) containing counter staining dye 4′,6-diamidino-2-phenylindole (Sigma) and viewed using a Zeiss Axiophot epifluorescent microscope. Images were captured with Compix imaging systems using a cooled RGB CCD camera and analyzed using Adobe PhotoShop 7.0. Cell counting was performed on digital images acquired from the slides. At least five randomly selected regions from different sections were counted for each group; and two-tailed, unpaired t-test was applied for statistical analysis. Data are expressed as mean±SEM.

### TaqMan real-time quantitative RT-PCR

TaqMan real-time quantitative PCR (RT-PCR) analysis was performed as described [45]. The following specific probes and primers were used for human Axin2 (probe, TGCCTCCTGGTCACACGAACAATGG; forward primer, CTCCAAGTGTCGTCCGGTTT; reverse primer, TGTACTCCGAGTCGGAATC), mouse Axin2 (probe, CCCAACGCGCCCTCTTTGATCTG; forward primer, TGGCTTTGACTACGCCCAC; reverse primer, GGGAGCTGAAGCGCTGG), rat Axin2 (probe TGTGAACAGTGCCAGGAGAGCGGT; forward primer, CCTGGATGTGCGGATTGC; reverse primer, TGCCCAGGAGCACTAGGC), and rat cyclin B2 (Applied Biosystems probe# Pr006906188.1). Probes and primers for the control housekeeping gene *Gapdh* were the same as reported [44]. Expression levels of genes of interest were normalized to *gapdh*. Initial RT-PCR amplifications were also examined by agarose gel electrophoresis to ensure that bands were only visible at the expected molecular weights.
